# Diabetes in* HFE* Hemochromatosis

**DOI:** 10.1155/2017/9826930

**Published:** 2017-02-26

**Authors:** James C. Barton, Ronald T. Acton

**Affiliations:** ^1^Southern Iron Disorders Center, Birmingham, AL 35209, USA; ^2^Department of Medicine, University of Alabama at Birmingham, Birmingham, AL 35294, USA; ^3^Department of Microbiology, University of Alabama at Birmingham, Birmingham, AL 35294, USA

## Abstract

Diabetes in whites of European descent with hemochromatosis was first attributed to pancreatic siderosis. Later observations revealed that the pathogenesis of diabetes in* HFE* hemochromatosis is multifactorial and its clinical manifestations are heterogeneous. Increased type 2 diabetes risk in* HFE* hemochromatosis is associated with one or more factors, including abnormal iron homeostasis and iron overload, decreased insulin secretion, cirrhosis, diabetes in first-degree relatives, increased body mass index, insulin resistance, and metabolic syndrome. In p.C282Y homozygotes, serum ferritin, usually elevated at hemochromatosis diagnosis, largely reflects body iron stores but not diabetes risk. In persons with diabetes type 2 without hemochromatosis diagnoses, serum ferritin levels are higher than those of persons without diabetes, but most values are within the reference range. Phlebotomy therapy to achieve iron depletion does not improve diabetes control in all persons with* HFE* hemochromatosis. The prevalence of type 2 diabetes diagnosed today in whites of European descent with and without* HFE* hemochromatosis is similar. Routine iron phenotyping or* HFE* genotyping of patients with type 2 diabetes is not recommended. Herein, we review diabetes in* HFE* hemochromatosis and the role of iron in diabetes pathogenesis in whites of European descent with and without* HFE* hemochromatosis.

## 1. Decreasing Prevalence of Diabetes and Cirrhosis in Hemochromatosis

**Figure 1:**
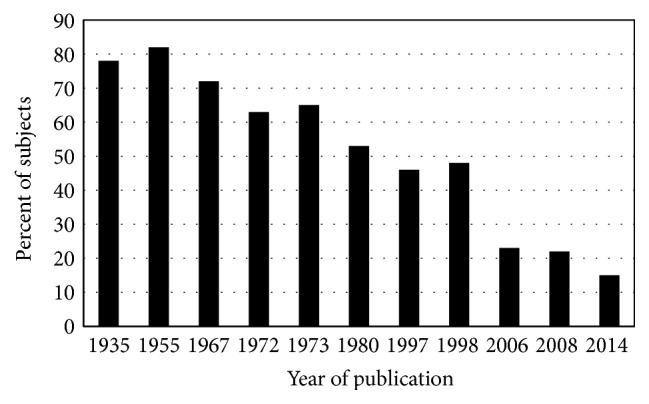
Diabetes in nonscreening hemochromatosis. Percentages of patients diagnosed to have hemochromatosis phenotypes in nonscreening settings who also had diabetes [1, 2, 8–10, 42, 131, 167–169].* HFE* mutation genotyping was a diagnostic adjunct in three studies [1, 2, 10].

**Figure 2:**
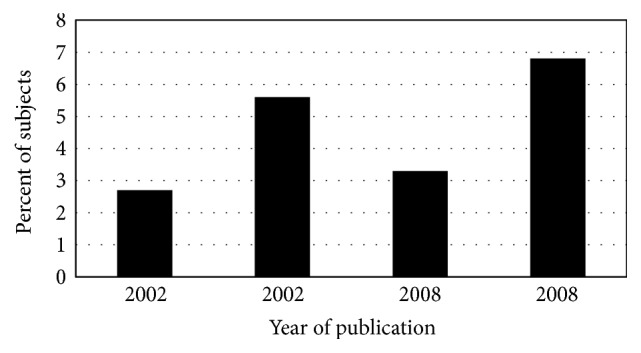
Diabetes in screening hemochromatosis. Percentages of participants in population-based screening studies discovered to have* HFE* p.C282Y homozygosity who reported that they had previous diagnoses of diabetes [115, 170–172]. There were two such reports from 2002 and two others from 2008. Hemochromatosis was also evaluated with iron phenotyping. In the respective populations, the prevalence of diabetes in p.C282Y homozygotes and control subjects did not differ significantly.

The prevalence of diabetes decreased among hemochromatosis case series published in the interval 1935–1998 (Figure 1). Earlier diagnosis of hemochromatosis due to iron phenotyping in probands and family members and subsequent phlebotomy therapy could partly explain the decrease. In two nonscreening hemochromatosis case series published in 2006 and 2008, respectively [1, 2], the prevalence of diabetes was lower than reported in the 20th C. The widespread adoption of* HFE* genotyping to confirm and enhance early hemochromatosis diagnoses after 1996 could explain much of the further decline in diabetes prevalence (Figure 1). The prevalence of diabetes in p.C282Y homozygotes identified in population screening programs is relatively low (Figure 2).

**Figure 3:**
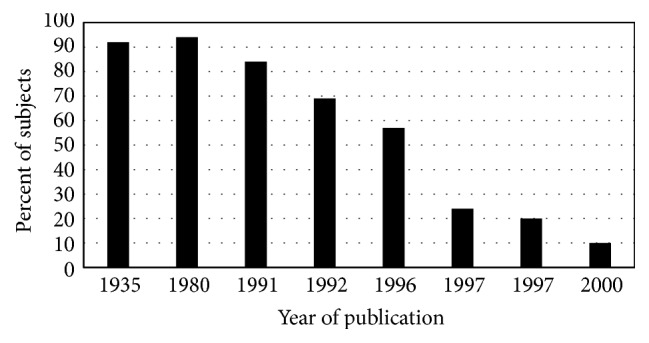
Cirrhosis in nonscreening hemochromatosis. Percentages of patients diagnosed to have hemochromatosis phenotypes who also had cirrhosis [3, 8, 169, 173–177]. There were two such reports from 1997.* HFE* mutation genotyping was a diagnostic adjunct in the more recent study [3]. Modified from [25, 178]. Greater proportions of men than women had cirrhosis. See cirrhosis prevalence in screening hemochromatosis cases in Figure 4.

**Figure 4:**
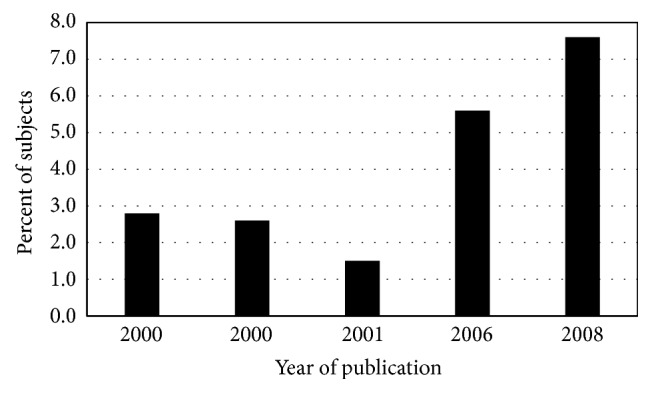
Cirrhosis in screening hemochromatosis. Percentages of participants in population-based studies [3, 172, 179, 180] and in an archived liver biopsy collection (second 2000 publication) [181] discovered to have* HFE* p.C282Y homozygosity who were previously diagnosed or were subsequently demonstrated to have advanced hepatic fibrosis or cirrhosis by biopsy [3, 170, 172, 180, 181]. Greater proportions of men than women had cirrhosis. See cirrhosis prevalence in nonscreening hemochromatosis cases in Figure 3.

The prevalence of cirrhosis in hemochromatosis case series also decreased in the interval 1935–1996 (Figure 3). Earlier diagnosis of hemochromatosis due to iron phenotyping of probands and family members and their subsequent phlebotomy therapy to achieve iron depletion could partly explain this decrease. The widespread adoption of* HFE* genotyping to enhance hemochromatosis diagnosis after 1996 could partly explain the further decline in cirrhosis prevalence in nonscreening hemochromatosis index patients reported in 2000 [3] (Figure 3). The proportion of p.C282Y homozygotes identified in population screening who had biopsy-proven cirrhosis was lower than that observed in nonscreening settings but is typically higher than in control subjects (Figure 4).

## 2. History of Hemochromatosis and Diabetes

In 1865, Trousseau described the syndrome of hepatic cirrhosis, pancreatic fibrosis, and cutaneous hyperpigmentation [4]. Troisier's confirmatory 1871 report of* diabète bronze et cirrhose pigmentaire* described iron deposition in various tissues [5]. In 1889, von Recklinghausen described staining excess iron and its tissue distribution at autopsy of persons with* hämochromatose* [6]. Clinicians in Europe and derivative countries reported the association of hemochromatosis and diabetes mellitus in whites with increasing frequency in the remaining 19th C [7]. Diabetes, a* sine qua non* of most hemochromatosis diagnoses through the first two-thirds of the 20th century, was usually observed in persons who also had severe iron loading and cirrhosis [8, 9].

During the latter third of the 20th century, the development of methods to measure serum iron, transferrin saturation (TS), and serum ferritin (SF) and the increased use of liver biopsy facilitated diagnosis of hemochromatosis phenotypes. After the discovery of* HFE* in 1996, hemochromatosis diagnosis shifted toward a genetic criterion. Most persons who were ascertained to have hemochromatosis using* HFE* genotyping had milder iron overload phenotypes and fewer complications, including diabetes, than patients with the diagnostic triad [10].

## 3. *HFE* Hemochromatosis

*HFE* hemochromatosis occurs as an autosomal recessive trait [11, 12] in 0.3–0.6% of whites of European descent [13–15] that is due to homozygosity for p.C282Y of the* HFE* gene (chromosome 6p21.3) [13].* HFE* is linked to the major histocompatibility complex (MHC) [13]. p.C282Y homozygosity accounts for ~90% of whites of European descent with “classical” hemochromatosis iron phenotypes [13–15]. Severe iron overload in p.C282Y homozygotes may cause cirrhosis, primary liver cancer, diabetes, other endocrinopathies, and cardiomyopathy [15, 16].

p.C282Y allele frequencies in whites who reside in different regions of Europe range from 13% in Ireland to less than 2% in Italy, Greece, and Spain [17–19]. p.C282Y allele frequencies in non-Hispanic whites who reside in North America are 6-7% [19]. Mean serum iron, TS, and SF levels of adults with p.C282Y homozygosity are higher than those of adults with other common* HFE* genotypes [20]. In clinical practice, persons with common* HFE* genotypes other than p.C282Y homozygosity cannot be distinguished by serum iron, TS, and SF measurements [19].

The membrane protein HFE has a structure similar to that of MHC class I proteins and also binds beta-2 microglobulin [13]. Transferrin receptor binds to the HFE extracellular *α*1-*α*2 domain [21, 22]. HFE contributes to regulation of hepatic synthesis of hepcidin, the main controller of iron metabolism [23].

Ferroportin, a transmembrane iron-binding protein and the hepcidin receptor, exports iron from absorptive enterocytes, macrophages, and hepatocytes [23]. Hepcidin regulates small intestinal iron absorption, plasma iron concentrations, and tissue iron distribution by inducing inactivation and ubiquitination of ferroportin [23, 24].

Hepcidin synthesis by the liver is regulated by extracellular and intracellular iron concentrations and the iron requirements of erythroid precursors via complex mechanisms that are incompletely understood [23]. In* HFE* hemochromatosis, excessive iron absorption and increased iron stores are due to lack of hepcidin upregulation, although mutations in non-*HFE* genes may act as positive “modifiers” of iron absorption in some p.C282Y homozygotes [19, 25, 26].

## 4. Measuring Iron Stores

Quantitative phlebotomy, the standard reference method for assessing body iron stores, permits measurement of the amount of iron mobilizable for hemoglobin synthesis [15, 27, 28] (Table 1). Measuring liver iron by atomic absorption spectrometry is also widely used for clinical assessments of iron overload [15, 29]. SF is the most widely used surrogate indicator of body iron stores (Table 1). Elevated SF levels in most patients without p.C282Y homozygosity are caused by noniron liver disease and other conditions [28, 30, 31] (Table 1). Histologic grading of Prussian blue positivity in bone marrow aspirates and liver biopsy specimens is not quantitative.

## 5. Diabetes Pathogenesis in Hemochromatosis

### 5.1. Pancreatic Siderosis

Through the mid-20th C, diabetes was observed in ~80% of patients with hemochromatosis (Figure 1). Most of them also had heavy liver iron loading and cirrhosis [8, 9]. Postmortem evaluations revealed severe hemosiderin deposition and iron-induced fibrosis of the islets of Langerhans and pancreatic acini [8, 9]. Specificity of iron deposition for the pancreatic beta cells was described in 1956 [32] and confirmed in 1987 [33].

### 5.2. Iron Entry into Pancreatic Islets

Transferrin receptors in normal human pancreas are expressed predominantly in the islets [34] and are presumed to be a physiologic means by which transferrin-bound iron enters islet cells. Divalent metal transporter 1 (DMT1) is also localized primarily to pancreatic islet cells [35]. The abundant expression of DMT1 in islet cells suggests that DMT1 also plays an important role in iron uptake by beta cells [36]. In a study of mice with global or tissue-specific inactivation of the* Slc11a2* gene that encodes DMT1, the investigators concluded that hepatocytes and most cells (other than placenta, small intestinal mucosa, and erythroid cells) “must have an alternative, as-yet-unknown, iron uptake mechanism,” although iron uptake by pancreas was not reported [37].

In* HFE* hemochromatosis, iron loading of parenchymal cells is partly due to uptake of non-transferrin-bound iron (NTBI) from plasma. In one study, an anti-DMT1 antibody significantly decreased the uptake of NTBI into human hepatocytes and hepatocellular carcinoma cells (HLF), although pancreas cells were not evaluated [38].

Mouse solute carrier Slc39a14 mediates NTBI uptake into cells in vitro [39]. Slc39a14 deficiency in mice with hemochromatosis induced by double homozygosity for* Slc39a14*^−/−^ and either* Hfe*^−/−^ or* Hfe2*^−/−^ greatly diminished liver iron loading and prevented iron deposition in hepatocytes and pancreatic acinar cells [40].

### 5.3. Hepcidin Expression in Beta Cells

Immunohistochemical studies in humans and rats localized hepcidin exclusively to pancreatic beta cells [41]. Immunoelectron microscopy analyses demonstrated that hepcidin is limited to the beta-cell secretory granules that store insulin [41]. Hepcidin expression in beta cells is regulated by iron in vitro [41]. Thus, beta cells, in addition to hepatocytes, are sources of hepcidin and may contribute to iron homeostasis and blood glucose regulation [41].

### 5.4. Decreased Insulin Secretion in* HFE* Hemochromatosis

In an early study, some patients with hemochromatosis had subnormal fasting plasma insulin levels and suboptimal increments in plasma insulin levels after intravenous glucose infusions [42]. In a subsequent report, loss of insulin secretory capacity was the primary event leading to hemochromatosis-related diabetes in thirty nonscreening patients with hemochromatosis (26 p.C282Y homozygotes) and mean SF 1501 ± 287 (standard deviation) ng/mL [1].

### 5.5. Iron, Islet Cells, and Insulin Secretion in* Hfe*^−/−^ Mice

Iron metabolism characteristics of* Hfe* knockout (*Hfe*^−/−^) mice are inherited as autosomal recessive traits and are typical of* HFE* hemochromatosis [19, 43], but iron metabolism characteristics of different* Hfe*^−/−^ mouse strains vary [44]. Hepatic gene expressions in* Hfe*^−/−^ mice profiles also differ by strain and age [45]. The inheritance of hepatic iron loading in* Hfe*^−/−^ mice is polygenic [46].

In* Hfe*^−/−^ mice (C57BL/6J genetic background) 10 weeks of age, Perls' Prussian blue-stained sections of liver, spleen, and small intestine, but not pancreas, revealed iron deposition [43]. At 10–12 months of age,* Hfe*^−/−^ mice (C57BL/6J background) had decreased glucose tolerance caused by inadequate increments of insulin levels [47].* Hfe*^−/−^ mice (129/SvEvTac background) had islet cell iron content that was 72% higher than that of wild-type controls by age 5 weeks [47]. Similar results were obtained in mice with homozygosity for a* Hfe* mutation knockin orthologous to human p.C282Y homozygosity [47]. Cooksey et al. concluded that excess iron in mice induces beta-cell oxidant stress and decreases insulin secretion due to desensitization and apoptosis [47]. Regardless,* Hfe*^−/−^ mice of both the C57BL/6J and 129/SvEvTac strains usually do not develop diabetes [47]. These observations suggest that diabetogenesis in* Hfe*^−/−^ mice requires decreased insulin secretion and other factor(s).

### 5.6. Diabetes and Liver Disease

Diabetes is a frequent complication of cirrhosis [25, 48–50]. Cirrhosis due to both iron overload and nonhemochromatosis causes occurs in* HFE* hemochromatosis [51]. Some persons with cirrhosis also have insulin resistance (IR) [52] and hyperglucagonemia [53] that may contribute to diabetogenesis. Kushner informally reported that “of 104 clinically affected male [hemochromatosis] probands, 32 (31%) had diabetes, and of these, 23 had biopsy-proven cirrhosis, five had moderate fibrosis, and only four had normal liver architecture” [47]. In hemochromatosis probands homozygous for p.C282Y diagnosed in medical care, neither biopsy-proven cirrhosis nor an aggregate variable “liver disorders” was significantly associated with diabetes [10]. In another study, the prevalence of diabetes in men with p.C282Y homozygosity and markedly increased iron stores (14% diabetes; 40% cirrhosis) did not differ significantly from that of men with p. C282Y homozygosity and normal or mildly increased iron stores (15% diabetes; no cirrhosis) [26].

### 5.7. Glucagon Secretion

Patients with hemochromatosis and impaired glucose tolerance or diabetes have enhanced glucagon responses after arginine infusion [54–59]. In one study, glucagon immunoreactivity in plasma was higher in patients with hemochromatosis than in control subjects, regardless of glucose tolerance [57]. When nonspecific reactivity was deducted, the resulting values for true glucagon concentrations were similar in hemochromatosis and control subjects [57]. It can be inferred from these reports that alpha-cell function is preserved in typical patients with hemochromatosis and diabetes [58].

### 5.8. Chromium

Retention of radiochromium administered intravenously to persons with hemochromatosis was reduced [60, 61]. Chromium potentiates the action of insulin in vivo and in vitro [62, 63] and may alleviate IR [64]. Chromium deficiency is common in persons with prediabetes [65]. Persons with type 2 diabetes have lower blood levels of chromium than those without diabetes [66]. DMT1 preferentially transports ferrous iron from the intestinal lumen into absorptive cells by a H^+^-dependent process. In* HFE *hemochromatosis,* DMT1* mRNA levels are increased [67]. Expression of DMT1 in* Xenopus* oocytes did not stimulate the transport of Cr(2+) or Cr(3+) [68]. Chromium, like iron, binds plasma transferrin [69, 70]. Mechanisms and kinetics of chromium absorption, intermediate metabolism, and excretion in hemochromatosis are unreported.

## 6. Iron Phenotypes and Diabetes

### 6.1. Hemochromatosis

The prevalence of previously undiagnosed hemochromatosis in patients attending a diabetes clinic in Australia was 2.4-fold higher than that of the general population [71]. The prevalence of hemochromatosis phenotypes was significantly greater in Italian patients with diabetes (117 type 1; 777 type 2) than control subjects (odds ratio 6.3) [72]. In the two aforementioned studies, hemochromatosis was diagnosed using iron phenotyping;* HFE* genotyping was not performed.

In the multiracial, multiethnic Hemochromatosis and Iron Overload Screening (HEIRS) Study of 97,470 participants in North America, 2.0% of participants who reported that they had diabetes also had hemochromatosis or iron overload [73].

### 6.2. Transferrin Saturation

TS was not a significant predictor of diabetes in non-Hispanic whites with p.C282Y homozygosity detected in the HEIRS Study [51]. In contrast, there was a significant negative trend of TS across increasing homeostasis model assessment-insulin resistance (HOMA-IR) quartiles in a postscreening cohort of p.C282Y homozygotes and* HFE *wild-type homozygotes [74].

In cohorts unselected for hemochromatosis, TS was inversely related to prediabetes [75] and diabetes [73, 76, 77]. In the HEIRS Study, mean TS was lower in non-Hispanic whites with diabetes [73]. To the contrary, TS was not a risk factor for diabetes in Australian adults in a large cross-sectional analysis [78]. In a meta-analysis, TS was a risk factor for type 2 diabetes [79].

### 6.3. Serum Ferritin in Diabetes with Hemochromatosis

SF levels were not significantly associated with diabetes in p.C282Y homozygotes identified in screening [51]. Neither SF nor quantities of iron removed to achieve iron depletion was significantly associated with type 2 diabetes in hemochromatosis probands with p.C282Y homozygosity diagnosed in medical practice [10]. In screening and nonscreening p.C282Y homozygotes, correlations of SF with iron burdens were positive and significant [51, 80]. These results indicate that increased storage iron in p.C282Y homozygotes, not diabetes, is the major determinant of SF levels [51].

### 6.4. Serum Ferritin in Diabetes without Hemochromatosis

SF levels were positively associated with fasting glucose [81]; impaired glucose metabolism [77]; insulin levels [81]; prediabetes [75]; and diabetes [73, 76–78, 82–85] in cross-sectional studies of participants unselected for hemochromatosis diagnoses. In a longitudinal study, SF levels were positively associated with glucose intolerance and IR [86]. In overall HEIRS Study analyses of observations of 97,470 participants, SF levels were significantly associated with diabetes in a regression model that included* HFE* genotype [73]. In 769 postscreening HEIRS Study participants (including 188 p.C282Y homozygotes), log SF was significantly associated with diabetes in a regression model that included* HFE* genotype [74]. In meta-analyses, there were positive associations of SF with type 2 diabetes [79, 87]. The ratio of serum transferrin receptor (sTfR) to SF (sTfR/SF ratio) was inversely associated with diabetes in case-control studies [82, 85, 88] and in a case-cohort study [84]. Lower ratios of sTfR/SF were independent predictors of incident type 2 diabetes [89]. Evidence from the EPIC-InterAct Study suggests that the relationship between type 2 diabetes and iron stores in persons unselected for hemochromatosis diagnoses is more complex than the association with SF levels alone [90].

### 6.5. Serum Ferritin in Diabetes: Hemochromatosis versus Nonhemochromatosis

Mean SF levels in subjects with untreated hemochromatosis and p.C282Y homozygosity [20, 91] were much higher than SF levels in subjects with or without diabetes who did not have* HFE* hemochromatosis genotypes [78, 92]. SF levels predict type 2 diabetes in persons without hemochromatosis diagnoses but the SF levels are typically below the concentration indicative of iron overload (7, 10, 13–22). Some authors mistakenly interpret or report higher mean SF levels in subjects with diabetes than controls or upward trends of SF levels across HOMA quartiles as evidence of increased body iron stores or iron overload. Elevated iron stores are not typical of patients with type 2 diabetes [88, 93]. A persistently elevated SF criterion has a low positive predictive value in screening patients with diabetes for hemochromatosis [94].

Hyperferritinemia is not significantly associated with diabetes in untreated p.C282Y homozygotes [10, 51]. In p.C282Y homozygotes, prephlebotomy plasma levels of C-reactive protein (CRP) and interleukin- (IL-) 6 did not differ significantly between those with high iron stores and those with low iron stores [95].

Ferritin is an iron storage protein. SF consists of iron-rich ferritin and iron-poor apoferritin [96, 97]. Body iron stores are in equilibrium with iron-rich SF [98, 99]. Levels and iron content of SF are increased in hemochromatosis and other iron overload disorders [30, 96]. The iron content of SF in noniron liver disorders associated with hepatocyte injury is also increased due to release of iron-rich hepatocyte ferritin into the blood [30, 96, 97]. Ferritin released from diverse tissues into the blood due to inflammation, anemia of chronic disease, or malignancy is typically apoferritin [30, 96, 97, 100–103]. Apoferritin synthesis and secretion are enhanced by interleukin-1 and chronic ethanol consumption [104, 105].

## 7. *HFE* Genotypes and Diabetes

In patients with type 2 diabetes, the prevalence of p.C282Y homozygosity did not differ significantly from that of control subjects [106–109]. The prevalence of undiagnosed diabetes or impaired fasting glucose in p.C282Y homozygotes identified in population screening was similar to that in control subjects with* HFE* wild-type genotypes [110]. Diabetes also occurs in some persons with the common* HFE* genotypes p.C282Y/p.H63D and p.H63D homozygosity [111, 112] and in other persons with hemochromatosis phenotypes and novel* HFE* genotypes [113, 114]. Regardless, clinical and screening studies of persons with hemochromatosis phenotypes did not detect significantly increased diabetes prevalence associated with common* HFE *genotypes, including p.C282Y homozygosity [2, 73, 115].

## 8. Morbidity and Mortality of Diabetes in* HFE* Hemochromatosis

### 8.1. Inflammation

Higher SF levels, lower TS levels, and higher blood neutrophil counts in patients with hemochromatosis and diabetes [51] may signify inflammation related to underlying processes that ultimately result in diabetes, rather than representing diabetogenic factors. Common inflammatory disorders in persons with diabetes, with or without hemochromatosis, include obesity, arthropathy, atherosclerosis, dyslipidemia, microvascular disease, and fatty liver. In persons with type 2 diabetes, CRP levels are elevated in ~50% of those with [51] and in ~40% of those without [116] hemochromatosis. Elevated CRP and IL-6 concentrations are significantly associated with increased type 2 diabetes risks in populations unselected for hemochromatosis diagnoses [117]. Subclinical inflammation is associated with hyperglycemia and IR in type 2 diabetes unassociated with hemochromatosis [118]. Single-nucleotide polymorphisms (SNPs) of three genes were associated with markers of islet cell inflammation [119].

### 8.2. Diabetes Risk

Decreased insulin secretion increases diabetes risk in persons with hemochromatosis [1, 42]. Obesity or increased body mass index (BMI) in persons with* HFE* hemochromatosis also increases diabetes risk [10, 51, 74, 120]. IR and metabolic syndrome (MetS) are common in patients with hemochromatosis [42, 121–123]. In non-Hispanic white adults without diabetes (including 188 p.C282Y homozygotes), IR as determined by HOMA-IR was a significant predictor of MetS but p.C282Y homozygosity was not [74]. In screening p.C282Y homozygotes, SF was significantly associated with HOMA-IR 4th quartile, MetS, and diabetes [51]. In addition, age, male sex, and BMI were significantly associated with HOMA-IR fourth quartile [51]. Only HOMA-IR fourth quartile was significantly associated with MetS [51]. Diabetes in first-degree family members was significantly associated with type 2 diabetes in hemochromatosis probands with p.C282Y homozygosity diagnosed in medical care (odds ratio 8.5 [95% confidence interval 2.9–24.8]) [10].

The general population prevalence of type 1 diabetes defined as autoimmune beta-cell destruction and absolute insulin deficiency is approximately the same as that of hemochromatosis [124]. Genes within the MHC are major risk factors for type 1 diabetes [125, 126], although diverse autoimmune conditions in 236 nonscreening hemochromatosis probands with p.C282Y homozygosity did not include type 1 diabetes [127]. In a population study of hemochromatosis and iron overload, it was unclear whether participants with “late-onset type 1 diabetes” had beta-cell autoimmunity [128]. Genome-wide association studies have not identified a consistent association of human leukocyte antigen (HLA) region genes with type 2 diabetes although many other associated genes occur on chromosomes other than 6p [129, 130]. Type 2 diabetes risk in nonscreening p.C282Y homozygotes was not associated with common HLA types and haplotypes [10].

## 9. Complications of Diabetes

### 9.1. Typical Complications

Many complications of diabetes in patients with hemochromatosis are typical of those that occur in patients without hemochromatosis. These include obesity; fat atrophy; proteinuria/albuminuria; retinopathy; peripheral neuropathy; and coronary artery and peripheral vascular disease [42, 131].

### 9.2. Diabetes, Arthropathy, Cirrhosis, and Pancreatic Cancer

The prevalence of second and third metacarpophalangeal arthropathy, a proxy for hemochromatosis hand arthropathy, was significantly associated with diabetes in p.C282Y homozygotes [51]. Erosive hand osteoarthritis in persons with type 2 diabetes unselected for hemochromatosis was also associated with hand pain [132]. Serum levels of the cellular adhesion molecule VCAM-1 were significantly associated with hemochromatosis arthropathy, independent of diabetes, BMI, and age [133]. Elevated VCAM-1 is also a significant predictor of incident diabetes [134]. Phlebotomy therapy reverses cirrhosis due to iron overload and hemochromatosis in some patients [135], although it is unreported whether cirrhosis reversal also reduces IR or diabetes manifestations. In a meta-analysis, the risk of cancer of the pancreas in persons with diabetes was increased (odds ratio 1.8) [136]. In contrast, pancreatic adenocarcinoma risk is not increased in hemochromatosis [137, 138], although adenocarcinoma of the pancreas has been described in hemochromatosis case series [10, 137, 138].

### 9.3. Diabetes, Survival, and Causes of Death in Hemochromatosis

Survival of German subjects after hemochromatosis diagnosis between 1959 and 1983 was decreased in those who had either cirrhosis or diabetes [139]. There was a 7-fold increased risk of death due to diabetes in patients with hemochromatosis [139]. The common feature of subjects with cirrhosis and diabetes was heavy iron overload [124, 139]. In a 1991 study of Canadian patients with hemochromatosis, diabetes did not increase the risk of death after data were controlled for the presence of cirrhosis [140]. In a large study of Danes reported in 2014, the mortality risk in individuals with diabetes was more than threefold greater in those with* HFE* p.C282Y/p.C282Y than in those with* HFE* wt/wt genotypes [141].

In the US, hemochromatosis was more likely to have been diagnosed in subjects who died with liver disease, liver neoplasms, cardiomyopathy, diabetes, or viral hepatitis [142]. The proportionate mortality ratios were even higher when liver neoplasms or liver disease was combined with diabetes [142]. Liver disease, liver neoplasms, cardiomyopathy, diabetes, and viral hepatitis were more likely to occur among hemochromatosis-associated deaths than among all deaths [142]. Men were more likely to have liver disease (excluding neoplasms), cardiac disorders, nonhepatic neoplasms, diabetes, liver neoplasms, and infectious diseases [142].

## 10. Iron in Nonhemochromatosis Diabetes

Most persons diagnosed to have type 2 diabetes do not have iron overload [88, 93]. Favorable effects of phlebotomy on diabetes manifestations suggest that abnormal distribution of normal quantities of body iron contribute to diabetogenesis in some persons without hemochromatosis [143–145]. Iron-related dietary, cellular, and molecular mechanisms may contribute to the development or expression of type 2 diabetes [146] (Table 2). These and other mechanisms may also be associated with or cause impaired glucose metabolism, IR, and MetS. It is plausible but unproven that the same mechanisms would be applicable to persons with hemochromatosis. Detailed review of these mechanisms is beyond the scope of this review.

## 11. Management of Diabetes in* HFE* Hemochromatosis

### 11.1. General Management

Treatment of type 2 diabetes in persons with or without hemochromatosis is similar [15, 147]. Reducing inflammation of diverse sources may have a positive effect on potentially injurious iron-related mechanisms, although this is unproven. In hemochromatosis probands with p.C282Y homozygosity, probands with diabetes had greater mean BMI [10, 120].

Physicians should recommend appropriate weight reduction via diet modifications and increased activity to all patients. Modifiable risks for patients with elevated CRP include suboptimal physical activity (men) and central obesity and lack of statin use (women) [116]. Some patients with diabetes would benefit from reduced consumption of red meat and alcohol [15]. Persons with hemochromatosis, diabetes, or chronic liver disease have increased risks to develop septicemia or wound infections due to* Vibrio vulnificus*, a cosmopolitan halophilic bacterium [148–152]. These persons should not consume uncooked shellfish or expose wounds to warm coastal waters [15, 148–152].

### 11.2. Phlebotomy

Persons with* HFE* hemochromatosis who present with elevated SF levels (men > 300 *µ*g/L, women > 200 *µ*g/L) should undergo phlebotomy therapy to achieve iron depletion [15, 147]. The goal of phlebotomy thereafter is to maintain nonelevated SF values [15]. Elevated TS in p.C282Y homozygotes is due to decreased hepcidin available to bind ferroportin and consequent increased storage iron release from macrophages and hepatocytes, not iron overload. Thus, elevated TS levels persist after iron depletion is achieved. Elevated TS is not a target of treatment in* HFE* hemochromatosis [15, 147].

In patients with hemochromatosis and diabetes who are presumed or known to have pancreatic siderosis, phlebotomy therapy is likely to improve insulin secretion only when hemochromatosis diagnosis and iron depletion are early [15, 153, 154]. Hemochromatosis patients with neither diabetes nor cirrhosis had normal insulin sensitivity but their acute insulin responses to glucose were decreased [122]. Phlebotomy treatment normalized their SF levels, increased their acute insulin responses, and normalized their glucose tolerance [122]. In five referred adults with hemochromatosis and iron overload, insulin secretory capacity improved after normalization of iron stores [155].

The efficacy of iron depletion in decreasing IR in persons with hemochromatosis or p.C282Y homozygosity is variable [122, 154, 156]. Phlebotomy therapy did not improve diabetes control in the majority of 44 patients with hemochromatosis (25 insulin-dependent, 19 noninsulin-dependent) [154]. In 15 men with hemochromatosis, phlebotomy therapy lowered insulin requirements in those with insulin dependency and improved diabetes control in about half of those without insulin dependency [154]. In another study, IR in patients with hemochromatosis and either cirrhosis or diabetes was unaffected by phlebotomy treatment [15]. Impaired glucose tolerance resulting from IR in hemochromatosis subjects with cirrhosis or diabetes is not affected by phlebotomy treatment [122].

Treating type 2 diabetes with phlebotomy is not routine. In type 2 diabetes without hemochromatosis, participants randomized to phlebotomy therapy achieved decreased hemoglobin A1c levels and favorable changes in insulin secretion and IR [143]. As expected, phlebotomy also decreased SF, TS, and hemoglobin levels [143]. Iron depletion improved control of poorly controlled type 2 diabetes in patients with elevated SF levels who did not have common* HFE* alleles [145]. Repeated phlebotomies of patients with type 2 diabetes significantly decreased serum glucose levels [144]. Blood donation or phlebotomy was associated with more favorable or improved metabolic indices associated with diabetes risk in subjects without diagnosed diabetes [144, 157–160]. In another study, blood donations did not influence diabetes risk [161].

## 12. Problems That Have Been Resolved

Pathogenesis of diabetes in* HFE* hemochromatosis is multifactorial and the clinical manifestations of diabetes are heterogeneous (Table 3). Increased type 2 diabetes risk in* HFE* hemochromatosis is associated with one or more factors, including iron overload, decreased insulin secretion, increased BMI, IR, MetS, diabetes in first-degree relatives, and cirrhosis. Iron overload alone is insufficient to cause type 2 diabetes in most p.C282Y homozygotes. Iron removed by phlebotomy is not significantly associated with diabetes in p.C282Y homozygotes in multivariate analyses. Phlebotomy therapy to achieve iron depletion does not improve control of diagnosed diabetes in all persons with* HFE* hemochromatosis. SF levels do not predict diabetes in p.C282Y homozygotes. No consistent association of chromosome 6p or HLA region genes (including* HFE*) that increase type 2 diabetes risk has been demonstrated. Prevalence of type 2 diabetes in persons with and without* HFE* hemochromatosis diagnosed today is similar. Routine iron phenotyping or* HFE* genotyping of patients with type 2 diabetes is not recommended. Persons with newly diagnosed type 2 diabetes who have arthropathy involving the second and third metacarpophalangeal joints are candidates for iron phenotyping or* HFE* genotyping because this manifestation is associated with increased diabetes risk. Iron overload alone is insufficient to cause type 2 diabetes in most* HFE* p.C282Y homozygotes.

## 13. Problems That Remain to Be Resolved

It is unknown whether phlebotomy of all p.C282Y homozygotes will increase insulin secretion if diagnosis of hemochromatosis and induction phlebotomy therapy are early. It is unknown whether early identification and treatment of p.C282Y homozygotes who also have common putative genetic “modifiers” that increase severity of iron phenotypes would decrease diabetes prevalence. Bone morphogenetic proteins have been implicated in glucose metabolism [162] and it has been proposed that* BMP2* rs235756 is associated with SF levels in p.C282Y homozygotes [163], although we found no documentation of the relationship of* BMP2* rs235756 to diabetes risk. It is unknown whether maintaining lower SF levels in persons with* HFE* hemochromatosis and diabetes than presently recommended for “maintenance” therapy could maintain or improve insulin secretion and diabetes control or decrease diabetes risk. The proportion of patients with* HFE* hemochromatosis who develop diabetes after diagnosis and treatment of hemochromatosis is unknown. The role of tumor necrosis factor (*TNF*; chromosome 6p21.33) in hemochromatosis-associated diabetes is unknown. The prevalence of* HFE* alleles and genotypes in cohorts of patients with autoimmune diabetes is unknown.

## 14. Directions for Future Research

Longitudinal studies that compare the incidence rates of diabetes in p.C282Y homozygotes and control subjects matched for age, sex, and race would provide greater insights into the burden of diabetes in* HFE* hemochromatosis, especially diabetes risk after hemochromatosis diagnosis. Effects of early diagnosis and phlebotomy on diabetes incidence in hemochromatosis could also be determined in longitudinal studies.

Genome-wide association or whole-genome sequencing studies of cohorts of p.C282Y homozygotes with and without diabetes could identify alleles associated with increased diabetes risk. It is anticipated that such studies would identify novel loci in p.C282Y homozygotes not previously identified in studies of participants with type 2 diabetes who were unselected for hemochromatosis diagnoses. Comparing frequencies of SNPs associated with* DMT1, SLC39A14, F13A1*,* RIPK2*,* STEAP4, *and* BMP2 *in p.C282Y homozygotes with and without diabetes would provide information about the role of these genes and their corresponding proteins in iron uptake in and inflammatory injury to beta cells.* TNF*-308G→A was significantly associated with TS but not SF levels measured in population screening [164]. Comparing frequencies of* TNF*-308G→A and other* TNF* promoter variants in p.C282Y homozygotes with and without diabetes would provide insights into the role of tumor necrosis factor in hemochromatosis-associated diabetes.* PCSK7* rs236918 genotyping in p.C282Y homozygotes may reveal relationships of cirrhosis risk [165] and changes in insulin sensitivity with dietary carbohydrate intake [166]. Analysis of* HFE* allele and genotype frequencies in patients with autoimmune diabetes and an appropriate comparator group would identify a significant relationship of common* HFE* alleles to autoimmune diabetes, if it exists.

## Figures and Tables

**Table 1:** Measures of body iron stores.

Measures	Specimen	Advantages	Disadvantages	References
Iron removed to achieve iron depletion	Blood	Standard reference method; therapeutic; minimally invasive; quantitative; whole body; widely available	Lengthy; inconvenient; moderate cost	[25, 27, 30, 182, 183]

Hepatic iron content	Biopsy	Invasive; quantitative; widely available; strong correlation with quantitative phlebotomy; permits evaluation of liver histology	Possible inadequate specimen; risks of pain, bleeding, pneumothorax, bile leak; single organ; moderate cost	[25, 183, 184]

Iron in liver	SQUID	Noninvasive; quantitative	Few devices exist; not routinely available; single organ; expensive	[183, 185–187]

Iron in liver, heart, pancreas	Magnetic resonance scan	Noninvasive; quantitative; detects iron overload over wide range of concentrations	Equipment expensive; all MRI devices not calibrated to measure iron	[183, 188]

Serum ferritin	Blood	Widely available; semiquantitative; inexpensive	Elevated in many subjects with excess alcohol consumption, inflammation, infection, chronic disease, malignancy; fair correlation with measured iron stores	[25, 28, 30, 80, 182, 183]

Serum transferrin receptor/serum ferritin (sTfR/SF)	Blood	Widely available; semiquantitative; inexpensive	Unsuitable for subjects with inflammation, infection, chronic disease, malignancy; not validated for iron overload study	[189, 190]

**Table 2:** Proposed roles of iron in type 2 diabetes.

Variable	Mechanism	Reference
Body iron status	Modulates transcription, membrane expression/affinity of insulin receptor expression in hepatocytes, influences insulin-dependent gene expression	[191]

Dietary iron	Controls circadian hepatic glucose metabolism through heme synthesis	[192]

Intake of processed meat, red meat	Higher risk of type 2 diabetes	[161, 193, 194]

Dietary iron restriction, iron chelation	Increased insulin sensitivity, beta-cell function (*ob/ob lep*^−/−^ mice)	[195]

Iron chelation	Ameliorates adipocyte hypertrophy via suppression of oxidative stress, inflammatory cytokines, and macrophage infiltration	[196]

Starvation	Increased liver *Pck1* transcription, hepcidin expression, and degradation of ferroportin; hypoferremia, hepatic iron retention (C57BL/6Crl, 129S2/SvPas, BALB/c, and *Creb3l3*^−/−^ null mice)	[197]

High fat diet	Increased hepatic iron regulatory protein-1, increased transferrin receptor 1 expression, increased hepcidin, decreased ferroportin (*Hfe*^−/−^ mice); increased fatty acid oxidation, hypermetabolism, elevated hepatic glucose production (*Hfe*^−/−^ mice)	[198, 199]

Cellular iron uptake	Stimulated by insulin	[200]

Excess hepatic iron	Hyperinsulinemia due to decreased insulin extraction, impaired insulin secretion	[121]

Iron-related proteins in adipose tissue	Expression modulated by insulin resistance	[201]

Adipocyte iron	Regulates leptin and food intake	[202]

Adiponectin	Transcription negatively regulated by iron	[203, 204]

Visfatin	Positive association with serum prohepcidin, negative correlation with serum soluble transferrin receptor in men with altered glucose tolerance	[205]

Heme oxygenase-1 promoter microsatellite polymorphism	Higher ferritin with short (GT)(*n*) repeats	[206]

Antioxidants	Lower levels partially explained by iron alterations	[207]

**Table 3:** Diabetes risk in *HFE* hemochromatosis.

Risk factors	Proposed mechanisms and pathophysiology
Increased iron entry into beta cells of islets	Increased transferrin saturation and transport via transferrin receptors Elevated nontransferrin bound iron in plasma and entry by incompletely described mechanisms Increased iron transport by divalent metal transporter 1

Decreased insulin secretion	Islet inflammation Beta-cell injury Pancreatic islet fibrosis

Cirrhosis	Associated with severe iron overload, pancreatic fibrosis Hyperglucagonemia *PCSK7* rs236918 allele C

History of diabetes in first-degree relatives	Multiple genetic and acquired factors

Genetic markers	Multiple loci for type 2 diabetes Chromosome 6p loci for type 1 (autoimmune) diabetes

## Abbreviations

BMI: Body mass index
CRP: C-reactive protein
DMT1: Divalent metal transporter 1
HLA: Human leukocyte antigen
HEIRS Study: Hemochromatosis and Iron Overload Screening Study
HOMA: Homeostasis model assessment
IL-6: Interleukin-6
IR: Insulin resistance
MetS: Metabolic syndrome
MHC: Major histocompatibility complex
NTBI: Non-transferrin-bound iron
SF: Serum ferritin
SNP: Single-nucleotide polymorphism
sTfR: Serum transferrin receptor
TS: Transferrin saturation.
